# Preventing Colorectal Cancer through Prebiotics

**DOI:** 10.3390/microorganisms9061325

**Published:** 2021-06-18

**Authors:** Manijeh Mahdavi, Isabelle Laforest-Lapointe, Eric Massé

**Affiliations:** 1 Department of Biochemistry and Functional Genomics, University of Sherbrooke, Sherbrooke, QC J1E 4K8, Canada; Manijeh.Mahdavi@usherbrooke.ca; 2 Department of Biology, University of Sherbrooke, Sherbrooke, QC J1E 4K8, Canada; isabelle.laforest-lapointe@usherbrooke.ca

**Keywords:** microbiome, microbiota, prebiotics, colorectal cancer, gut dysbiosis, short chain fatty acids, inflammation, cancer prevention

## Abstract

Colorectal cancer (CRC), the third most common cancer in the world, has been recently rising in emerging countries due to environmental and lifestyle factors. Many of these factors are brought up by industrialization, which includes lack of physical activity, poor diet, circadian rhythm disruption, and increase in alcohol consumption. They can increase the risk of CRC by changing the colonic environment and by altering gut microbiota composition, a state referred to as gut dysbiosis. Prebiotics, which are nutrients that can help maintain intestinal microbial homeostasis and mitigate dysbiosis, could be beneficial in preventing inflammation and CRC. These nutrients can hinder the effects of dysbiosis by encouraging the growth of beneficial bacteria involved in short-chain fatty acids (SCFA) production, anti-inflammatory immunity, maintenance of the intestinal epithelial barrier, pro-apoptotic mechanisms, and other cellular mechanisms. This review aims to summarize recent reports about the implication of prebiotics, and probable mechanisms, in the prevention and treatment of CRC. Various experimental studies, specifically in gut microbiome, have effectively demonstrated the protective effect of prebiotics in the progress of CRC. Hence, comprehensive knowledge is urgent to understand the clinical applications of prebiotics in the prevention or treatment of CRC.

## 1. Introduction

Colorectal cancer (CRC) is classified as the third most commonly diagnosed cancer in men and the second in women worldwide [1]. CRC is the fourth major cause of cancer-related deaths, and its incidence and mortality rate is expected to rise by 60% to over 2.2 million new cases and 1.1 million deaths by 2030 [2]. This growth is hypothesized to be the product of several factors such as economic development, environmental changes, more sedentary lifestyle, obesity, alcohol, processed food and meat consumption, and increased longevity [3]. It is important to note that over the past decades, the incidence of early-onset CRC, which is generally defined as CRC diagnosed in populations less than 50 years old, is increasing at an alarming rate in the entire world [4,5]. While the reasons for this global increase are unclear [6], lifestyle factors such as diet may be the major contributing factor in comparison to genetic changes or mutations, which account for a smaller proportion of cases [7].

Diet plays a key role in both prevention and development of CRC [8,9]. Indeed, based on the conclusions of a recent meta-analysis, there is a striking correlation between meat consumption and CRC rates [10]. Both processed and unprocessed red meat increase the risk of CRC by pathologically changing the colonic environment, including gut microbiome composition, thus mediating a key transition from colonic homeostasis to colonic dysbiosis. This state of colonic perturbation can activate pro-tumorigenic immune signaling cascades, leading to pro-carcinogenic inflammation, carcinogen production, and transformed cellular reactions in vulnerable hosts, resulting in the progression of CRC [11]. However, high consumption of dietary fiber, considered as prebiotics, has been associated with reduced risks of CRC, suggesting a protective effect of these molecules [12,13]. Although the overwhelming body of evidence is in favor of the beneficial effects of prebiotics, some studies have reported a pro-tumorigenic effect of oligosaccharides, e.g., inulin, in some animal models and in in vitro studies [14].

Prebiotics are recognized as non-digestible food components conferring health benefits associated with modulation of the host’s gut microbiota, which are referred to as probiotics [15]. Prebiotics may be classified based on the number of monomers bound together, such as disaccharides, oligosaccharides (3–10 monomers), and polysaccharides. The most favorable criteria for the classification of prebiotic materials are oligosaccharides, comprising: xylooligosaccharides (XOS), fructooligosaccharides (FOS), isomaltooligosaccharides (IMO), transgalactooligosaccharides (TOS), galactooligosaccharides (GOS), and soybean oligosaccharides (SBOS) [16,17]. Additional dietary polysaccharides such as cellulose, hemicellulose, starch, inulin, or pectin may possibly be prebiotics [18].

There is an increasing body of evidence describing the preventive effect of prebiotics on CRC. In this review, we highlight recent research performed in mice, rats, and humans describing successful and unsuccessful use of prebiotics. We discuss potential mechanisms of prebiotics conferring colorectal cancer prevention with respect to their effects on gut microbiome structure and microbial metabolite production in the colon environment.

## 2. Prebiotics and CRC

The efficacy of prebiotics in CRC is well supported by animal studies. Recent research in Apc^Min/+^ mice, have shown that a diet containing the prebiotic triterpenoid saponins from *Gynostemma pentaphylum* (GpS) results in a considerably reduced number of polyps in the colon achieved through the mutualistic interaction between a probiotic, *Bifidobacterium animalis*, and triterpenoid saponins [19]. Another recent study reported that jujube polysaccharides as a prebiotic had protective effects against colorectal cancer induced by azoxymethane/dextran sodium sulfate (AOM/DSS) in C57BL/6 mice [20]. In other studies on mice, similar results were obtained through the prebiotic effects of the polysaccharides from a cyanobacterium called *Nostoc commune* Vaucher (NVPS) [21], acacia gum [22], Mushroom *Ganoderma lucidum (Lingzhi)* polysaccharides (GLP) along with GpS [23], and 50% chitin-glucan with raw potato starch [24] (see Table 1 for a summary). In Wistar rats with induced CRC, Yacón flour as a source of fructooligosaccharides may help to maintain the integrity of their intestinal health [25]. Similarly, this prebiotic, along with the commercial probiotic VSL#3^®^ (Sigma-Tau Pharmaceuticals, Gaithersburg, MD, USA) showed extra benefits in C57BL6/J mice, in comparison with the use of VSL#3^®^ alone, culminating in a significant reduction in precursor lesions of CRC [26]. In other new researches on rats developing CRC, either genetically or by induction carcinogens such as AOM/DSS, various prebiotics such as galacto-oligosaccharides derived from lactulose [27], inulin [28], phenolic compounds such as anthocyanins and ellagic acid from *Myrciaria jaboticaba* ((Vell.) O.Berg) seeds [29], and Djulis (*Chenopodium formosanum*), a native cereal crop [30], had preventive effects against CRC progression (Table 2). Notably, the positive impact of prebiotics on CRC progression can be seen in various human colon cell lines via the use of fructo-oligosaccharides (FOS) [31], soluble dietary fiber extracted from plantain inflorescence [32], and polysaccharide fraction from mushrooms *Cantharellus cibarius* [33], respectively (Table 3).

Although animal models strongly supported the favorable effects of prebiotics on CRC burden, human based studies are still limited [34]. In one clinical trial study involving 140 perioperative patients with CRC (90 men and 50 women, aged 40–75 years) in China, oral intake of 30 g prebiotic supplement (Hangzhou Niuqu Biotech Co., Hainengbo, China) containing fructooligosaccharide (25%), xylooligosaccharide (25%), polydextrose (25%), and resistant dextrin (25%) showed significant effects on immunologic indices in both the preoperative and postoperative periods of patient with CRC. Furthermore, prebiotics changed the abundance of four commensal microbiota (*Bacteroides*, *Bifidobacterium*, *Escherichia-Shigella*, and *Enterococcus*), and opportunistic pathogens in these patients [35]. Although these studies show positive effects of prebiotics in clinical settings (Table 4), other studies do not support the use of prebiotic supplements to diminish the risk of CRC mortality amongst postmenopausal women [36]. In fact, they investigated the influence of prebiotic fiber supplements categorized as soluble and insoluble in a cohort study including 160,195 postmenopausal women in the United States [36]. These controversial data suggest that more research is necessary to be done to completely explain their clinical impact in reducing CRC burden at population-based levels.

## 3. Mechanisms of Prebiotics in CRC Prevention

### 3.1. Modulation of Gut Microbiome and Maintaining a Microbial Homeostasis

The definition of prebiotics by the International Scientific Association for Probiotics and Prebiotics (ISAPP) is “a selectively fermented component allowing particular modifications, both in the composition and/or activity in the gastrointestinal microbiota which provides benefits upon host well-being and healthiness” [37]. The use of prebiotics should increase the proliferation of one or a limited number of bacteria in the colon and their specific metabolites, which may have a valuable effect on anti-cancer treatments [38]. Prebiotics can exert health beneficial effects on the colon by being metabolized by specific bacteria [39]. These nutrients drive the decline or growth of certain bacterial groups depending on the type of prebiotic [40]. Studies indicated that inulin-rich foods can enhance prominent propionate producers in *Bacteroidetes* populations, mainly due to a significant increase in the *Bacteroidaceae*, *Porphyromonadaceae*, and especially *Prevotellacea* families [28]. Inulin also helps to reduce the phylum *Firmicutes*, primarily due to lower *Lachnospiraceae* populations. It should be noted that high ratios of *Firmicutes*/*Bacteroidetes* are mostly related to inflammation-associated diseases, such as obesity or diabetes [41,42]. Therefore, the lower ratio demonstrated in this study indicated a protective effect of inulin. In addition, consumption of inulin led to a substantial reduction in pro-inflammatory bacterial populations, such as those related to the genus *Desulfovibrio* and *Bilophila* [28]. Furthermore, in a recent research on rats with induced CRC, prebiotic phenolic compounds such as anthocyanins, ellagic acid, and ellagitannins were able to restore the biodiversity of bacteria in all groups by changing the abundance of *Firmicutes*, *Bacteroidetes*, and *Proteobacteria* phyla [29]. Other prebiotics with such characteristics in suppressing harmful bacteria and maintaining colonic microbial homeostasis are summarized in Table 1, Table 2, Table 3 and Table 4.

Some of the key prebiotics that are most easily processed by the intestinal microbiota are particular sugars identified as oligosaccharides, which can be classified as non-digestible oligosaccharides (NDO). These oligosaccharides have special glycosidic bonds of the anomeric carbon atoms from the monosaccharide unit that cannot be broken down by the human gastrointestinal tract enzymes [43]. The most common of the NDOs, Fructooligosaccharides (FOS), galactooligosaccharides (GOS), and Xylooligosaccharides (XOS) affect microbiota composition [44], resulting in a higher number of intestinal *Bifidobacterium spp.* and *Lactobacillus* spp. [45,46,47,48,49]. Moreover, Fernández, J. et al. (2018), reported Galacto-oligosaccharides, derived from lactulose, accounted for increased *Bacteroidetes* and *Bifidobacterium* and reduced *Firmicutes* populations. Their research also demonstrated higher number of other good propionate producers such as *Paraprevotella* and *Parabacteroides* genus, both of which are associated with health benefits including protection against pro-inflammatory gut conditions and CRC [27].

### 3.2. Production of Fermentation Metabolites: SCFAs

Many of the beneficial effects of microbial alterations are mediated by prebiotics’ metabolites, such as short chain fatty acids (SCFAs) [50]. However, some prebiotics such as jaboticaba (*Myrciaria jaboticaba*) seed extract have phenolic compounds of ellagic acid and ellagitannins that can be degraded in the gut into urolithins. The compound urolithin was shown to inhibit the propagation of cancer cells by regulating signaling pathways and cellular activities and minimizing the stimulation of proinflammatory cytokines in the gut [51].

Among the fermentation products of prebiotics from the microbiota, SCFAs are considered the most important [52]. SCFAs are small molecules made by certain types of bacteria such as *Bifidobacterium* spp. and *Lactobacillus* spp. that critically impact colonocytes’ metabolism, cellular metabolism, host immune response and health, signalling pathways, epigenetics, and gene expression through multiple mechanisms [50,53,54]. Fermentation of prebiotics by the gut bacteria produces many metabolites and gases including SCFAs, whose most striking compounds are butyrate, propionate, and acetate [50,52]. These can be used as energy sources absorbed by the colonic mucosa and help colonocytes maintaining the protective mucosal barrier [55]. While acetate is metabolized by muscle, the kidneys, heart, and brain for gluconeogenesis (synthesis of glucose from non-carbohydrate), propionate is a neoglucogenic substrate that may inhibit cholesterol synthesis and regulate lipogenesis in adipose tissue [56]. Conversely, butyrate is metabolized by colonic commensal bacteria, where it plays a critical role as a preferential substrate and control cell differentiation by various mechanisms discussed below [57]. As prebiotics produce a variety of SCFAs from microbiota fermentation, specific prebiotic fibres should be selected for the treatment of inflammatory diseases [58]. For example, Yacón flour as a source of fructooligosaccharides in animals with induced colorectal carcinogenesis induces greater production of acetic, propionic, and butyric acids, and total SCFAs [25]. In addition, inulin-rich foods give rise to the in situ production of SCFAs such as propionic and butyric acids [28] or lactulose, thus causing a significant increase in the production of propionate [27]. Furthermore, there is compelling evidence for the release of butyrate from acacia gum consumption [22], and a higher production of acetate along with butyrate by using NVPS [21] and chitin-glucan [24]. Lastly, SCFAs are also responsible for many important physiological functions, including preserving the luminal pH, stopping pathogen growth, influencing the bowel motility, and reducing colon cancer by inducing cancer cells apoptosis [59].

#### 3.2.1. G-Protein Coupled Receptors (GPCRs)

Besides serving as energy source, SCFAs also act as ligands that bind specific G-protein coupled receptors (GPCRs) on colonocytes and immune cells [60]. As such, they can act as signalling molecules to decrease the production of proinflammatory cytokines and increase the total number of regulatory T (Treg) cells in the large intestine, through GPCRs [61]. GPCR43 (FFAR2), GPCR41 (FFAR3), and GPCR109A are the main GPCRs that bind specifically to SCFAs. Whereas both GPCR41 and GPCR43 can bind to butyrate, propionate, and acetate, GPCR109A seems to be more specific to butyrate [60]. Most anti-carcinogenic modifications in the gut microbiota are caused by these receptors [24,62,63,64]. For example, SCFAs stimulates GPCR43 on regulatory T cells, activating their expansion and preventing procarcinogenic inflammation [65]. Additionally, Bishehsari et al. (2018) demonstrated that when butyrate levels increased, in colon specific polyposis mice treated by 50% chitin-glucan and 50% raw potato starch as a prebiotic, the GPCR109A expression was boosted, and the tumour counts were reduced [24].

#### 3.2.2. Epigenetic Effects

SCFAs can inhibit carcinogenesis through several mechanisms. One of these mechanisms is the induction of histone modification, which leads to suppression of NF-κB signalling in cells [66,67,68,69,70,71]. These variations can have many effects depending on the genes affected [66,67,68,70,71]. For example, one study showed that butyrate induces cellular apoptosis in colon cancer cell lines and prevent their growth by increasing p57 expression [32,68]. Indeed, butyrate increases p57 mRNA transcription through inhibition of a histone deacetylase (HDAC) activity [68]. Thus, butyrate has been proposed to exhibit positive effects on CRC patients by prompting CRC apoptosis, reducing inflammation, modulating oxidative stress, and improving epithelial barrier function [72].

Regulation of MUC4 expression is another epigenetic illustration of SCFAs hindering colorectal carcinogenesis. Studies have shown that butyrate decreases the expression of HNF-4α in colon cancer cell lines which, in turn, reduces the expression of MUC4. Mucins, encoded by the MUC genes, mediate tumour interactions with immune cells, encouraging cellular proliferation and metastasis. Due to its role as a ligand to the receptor tyrosine kinase ErbB2, MUC4 has been of particular interest [66]. Lastly, a unique mechanism of the epigenetic effects of SCFAs is the impact of butyrate on spleen tyrosine kinase (Syk), a non-receptor tyrosine kinase that plays a pivotal role in cancer progress [73]. SCFAs also inhibit COX-2 enzyme and thus decrease prostaglandin production [30]. Together, they help increase apoptotic activity and decrease the proliferation of tumour cells, while allowing normal cells to proliferate [9].

### 3.3. Direct Effects of Prebiotics

Prebiotics possess other properties, such as the modification of gene expression in bacterial cells in cecum, the colon, and feces; enhancement of absorption of micronutrients in the colon; and the modulation of xenobiotic-metabolizing enzymes [74,75]. Acacia gum, a soluble fiber prebiotic, significantly reduced the levels of β-glucuronidase in mice with induced CRC [22]. Fidelis M et al. (2021) reported polyphenol ingredients from jaboticaba seed extract including castalagin, vescalagin, procyanidin A2, and ellagic acid can decrease bacterial metabolizing enzymes such as β-glucosidase, mucinase, β-glucoronidase, β-galactosidase, and nitroreductase, which results in reduced colonic cancer incidence. In addition, polyphenols can prevent the proliferation of *Clostridium, Bacteroides*, and *Propionibacterium* spp., thus enhancing the abundance of beneficial *Bifidobacterium* and *Lactobacillus* species. The plausible mechanism for this antimicrobial effect of polyphenols could be directed by hydrogen bonding of their hydroxyl groups to lipid bilayers of cell membranes, and also intercalation or hydrogen bonding with nucleic acid bases of RNA and DNA. These mechanisms consequently inhibit bacterial growth and chelating iron ions in the gut, creating an unwelcoming environment for the growth of aerobic microorganisms, principally gastropathogenic bacteria [29].

Other studies stated that around 10% of phenolic constituents are bioavailable and the remaining parts are cleaved by intestinal microbiota into other low-molecular-weight phenolic substances that can either be modulated or absorbed by the microbiota [76]. Yacón flour is also a source of phenolic acids, mainly caffeic and chlorogenic acids, both of which work as antioxidant that helps reduce oxidative stress [77]. Moreover, Yacón flour can increase epithelial mucus production and maintain the integrity of intestinal tight junctions that prevent bacterial translocation [78]. In addition, prebiotics such as oligosaccharides can interact with the bacterial receptor by imitating the microvillus glycol-conjugates and then prevent pathogens from attaching to epithelial cells, effectively inhibiting pathogen colonization [79,80].

Prebiotics such as jujube polysaccharides showed they can affect certain metabolic pathways contributing to host health, such as key pathways involved in metabolism, ATP-binding cassette (ABC), and two-component system transporters, and ABC transporters, including those predicted to be involved sugar and amino acid metabolism [20]. Similarly, genes of six key pathways were expressed differentially in colorectal cancer mice consuming NVPS. These pathways include metabolic processes related to amino acids, cofactors, vitamins metabolism, glycan biosynthesis and metabolism, and biosynthesis of other secondary metabolite pathways responsible for cellular processes and signaling [21]. Prebiotics are also assumed to be directly absorbed into colonic cells and change the host gene expression profile. Using oligosaccharides with different degrees of polymerization (DP), a research study has validated that only prebiotics with low DP can amplify IL-10 and IFN-γ production in CD4+ T cells, suggesting its integral uptake through the colon and then modulation of intestinal immune response [81].

### 3.4. Immunomodulation

Prebiotics maintaining a healthy gut microbiota can ensure both immune defense and prevention of diseases, such as CRC, by decreasing cell proliferation, stimulating the induction of apoptosis, inhibition of angiogenesis, and delay of the metastatic process [82,83]. Prebiotics such as Yacón may have a direct immunomodulation effect and increase the levels of sIgA. The increase in sIgA levels is attributed to the FOS contents of Yacón fermented in the cecum by members of the genus *Bifidobacterium* [84]. In addition, Yacón supplementation has a significant effect on immune system which is evidenced by a lower ratio in the TNF-α/IL-10 ratio, representing a balance between pro- and anti-inflammatory cytokines. IL-10 is produced by Th2 lymphocytes and inhibits macrophage dependent cytokines synthesized by Th1 cells that also produce TNF-α [85]. Thus, an auto-regulatory loop seemingly exists in which TNF-α stimulates IL-10 production, which, in turn, reduces TNF-α synthesis [86]. BALB/c mice supplemented with a Yacón-based product, which included FOS, showed an increase in the percentage of regulatory T cells (T reg) in the colon, and these cells also produce IL-10. Generally, immune system modulations are observed with greater production of antibacterial defensins, sIgA, and anti-inflammatory cytokines, mainly IL-10 [25].

In a clinical study of patients with CRC, prebiotic supplementation significantly increased IgG and IgM levels preoperatively [35]. However, postoperatively the supplementation enhanced the levels of IgG, IgA, total B lymphocytes (CD19+), and suppressor/cytotoxic T cells (CD3+CD8+). The use of prebiotics increased the level of transferrin, which relieved the inflammatory reaction of the body. The authors of this study concluded that prebiotic intake is recommended to improve serum immunologic indicators in patients with CRC a week before operation [35].

Moreover, NVPS could activate macrophages in vitro to suppress colorectal cancer [87]. The mushroom *Ganoderma lucidum* (Lingzhi) polysaccharides (GLP), along with saponins extracted from *Gynostemma pentaphyllum* (GpS), an herbal tea, clearly improved the inflamed gut barrier of mice. This effect is mediated via reducing polyps, shifting colonic M1 to M2 macrophages, positively reverting E-cadherin/N-cadherin ratio, and down regulating oncogenic signaling molecules [23].

## 4. Conclusions

The use of prebiotics is a promising therapy strategy which is safe in different clinical settings. High fiber supplementation including prebiotics modifies a microbiota community significantly, increases SCFA-producing bacteria, amplifies functional pathways related to SCFA, and raises the levels of SCFA metabolites. Notably, most studies have shown that an increase in SCFAs is relevant to a significant decrease in tumor loads. Benefits from consumption of prebiotics include antimicrobial activities against gut pathogens, modulation of the immune system, reducing gut inflammation and colitis, prevention of CRC, gut homeostasis, and regulation of the host energy metabolism. Future prospects indicate that the intestinal microbiota can be enriched and regulated by the addition of prebiotics into the diet, with special emphasis on biologically active compounds existing in foods of plant origin and that can mitigate or attenuate the CRC development.

## Figures and Tables

**Table 1:** Prevention of Colorectal cancer (CRC) Using various prebiotics in mice.

Reference	Type of Study	Prebiotic	Probiotic	Mechanism of Action
[19]	Research In Apc^Min/+^ mice	Triterpenoid saponins from *Gynostemma pentaphylum*	*Bifidobacterium animalis*	✓ Suppressed potential harmful bacteria, such as sulfur reducing bacteria. ✓ Promoted the SCFA producing bacteria. ✓ A potent anti-inflammatory and anticancer properties in Apc^Min/+^ mouse models. ✓ The modulation of the gut commensal bacteria. ✓ GpS is well served as the growth stimulus to *B. animalis* through the activations of a series of genes encoding for rRNA and various biogenesis protein molecules. ✓ Reduced polyp burden in Apc^Min/+^ mice. ✓ Production of short-chain and medium-chain fatty acids. ✓ Regulated biogenesis and metabolic pathways.
[20]	Research AOM)/DSS ^1^-induced CRC C57BL/6 mice	Jujube polysaccharides (JP) Chines fruit		✓ Significant protective effects against CRC, and a strong activity in regulating dysbiosis and maintaining a balanced microbial ecology. ✓ There was a significant decrease in *Firmicutes*/*Bacteroidetes* post JP treatment. ✓ Positively modulated intestinal microbiota and affecting certain metabolic pathways contributing to host health. ✓ Could cause variations in specific microorganism populations, whereas microbial conversion of polysaccharides affects other colonic pathways and processes, such as SCFA production. ✓ JP intervention affected cellular component genes, including those in the cytosol, cytoplasm, plasma membrane, membrane, and integral components of membrane, to relieve the negative consequences AOM/DSS induction.
[21]	Research AOM/DSS-induced CRC C57BL/6J mice	the polysaccharides from *N. commune* (NVPS) ^2^		✓ Enhanced immune activity of microenvironment in intestinal tract. ✓ Alleviated this malignancy to some degree, including reduced number and size of tumor and decreased expression of markers of CRC. ✓ Community composition of gut microbiota was modulated as a whole at the phylum and genus levels after treatment with NVPS. The data showed NVPS were able to reverse the microbiota community shift caused by AOM/DSS in group model, of which 6 phyla (*Firmicutes*, *Bacteroidetes*, *Proteobacteria*, *Verrucomicrobia*, *Cyanobacteria*, and *Actinobacteria*) and 57 genera were affected. ✓ SCFA-producing bacteria are the main action microorganism of NVPS on colon tumorigenesis in mice, indicated by impacts of NVPS on the population and metabolites of bacteria in the intestine. ✓ NVPS can regulate the metabolic process and reshape the metabolism mode of gut microbiota in colorectal cancer mice, which may be an important pattern of NVPS-induced inhibition against colon tumorigenesis by modulating gut microbiota. ✓ Activated macrophages in vitro to suppress colorectal cancer.
[22]	Research BALB/C mice	Acacia gum ^3^	(*Lactobacillus plantarum* MBTU-HK1)	✓ TNF-α levels were significantly reduced due to anti-inflammatory activity of acacia gum. ✓ The fermentation of acacia gum by colonic bacteria releases butyrate. Butyrate serves as a potent anti-inflammatory agent by inhibiting NFKB. ✓ Reduction in the levels of β-glucuronidase.
[23]	Research Apc^Min/+^ mice	Mushroom *Ganoderma lucidum* (Lingzhi) polysaccharides (GLP) along with the saponins extracted from *Gynostemma pentaphyllum* (GpS), an herbal tea		✓ Profoundly improved the inflamed gut barrier of Apc^Min/+^ mice by reducing polyps, shifting colonic M1 to M2 macrophages, positively reverting E-cadherin/N-cadherin ratio, and down-regulating oncogenic signaling molecules. ✓ Promoted short-chain fatty acids SCFAs-producing bacteria and abridged sulfate-reducing bacteria in a time-dependent manner. ✓ G-protein coupled-receptors were significantly stimulated in the treated mice, accompanied by the modulated expressions of histone deacetylases, anti-cancer gut hormone PYY, and PPAPγ. ✓ Modulated the relationship between the host and the gut microbiota.
[24]	Research TS4Cre × cAPC^l◦×468^ mice	50% chitin-glucan (KitoZyme SA, Herstal, Belgium) and 50% raw potato starch, prepared at Purdue University, West Lafayette, IN, USA		✓ Changed microbiota population significantly. ✓ Increased SCFA-producing bacteria. ✓ Augmented SCFA-related functional pathways. ✓ Elevated the levels of SCFA metabolites. ✓ Increased the relative abundances in both acetate-producing bacteria *Bifidobacterium* and both butyrate-producing clostridial cluster XIVa bacteria *Lachnospiraceae* unclassified and *Anaerostipes*. ✓ *Lachnospiraceae* unclassified and *Anaerostipes* are believed to benefit the host’s gut barrier functions through anti-inflammation, anti-tumorigenesis, and pathogen exclusion in the colon. ✓ Increased SCFA-producing bacteria resulted in the augmentation of butanoate and propanoate metabolism pathways upon high fiber treatment.

^1^ Azoxymethane/Dextran Sodium Sulfate, ^2^ *Nostoc commune* Vaucher (*N. commune*), a macroscopic cyanobacterium, ^3^ is a soluble fiber used as a food additive.

**Table 2:** Prevention of Colorectal cancer (CRC) using various prebiotics in rats.

Reference	Type of Study	Prebiotic	Probiotic	Mechanism of Action
[25,26]	Research Wistar rats	Yacón flour-fructooligosaccharides	-	✓ Reduced pH. ✓ Intestinal permeability: prebiotics affect intestinal barrier integrity by increasing epithelial mucus production and maintaining the integrity of tight junctions that prevent bacterial translocation. ✓ Total antioxidant capacity. ✓ The TNF-α/IL-10 ratio. ✓ SCFAs with greater production of acetic, propionic, and butyric acids. ✓ Immune system modulation is observed with greater production of antibacterial defensins, sIgA, and anti-inflammatory cytokines, mainly IL-10.
[28]	Research 2 model Rats 1-Develop colon cancer by carcinogen azoxymethane (AOM) and dextran sodium sulfate (DSS). 2-genetic mutation in APC gene	Inulin-rich foods 15.7% in chorizo and 10% in cooked ham		✓ Enhanced beneficial colon microbiota populations, giving rise to the in situ production of short-chain fatty acids (SCFAs) such as propionic and butyric acids. ✓ Significantly increased *Bacteroidetes* populations mainly due to an increase in *Bacteroidetes* and *Prevotellaceae* families, together with a reduction in *Firmicutes*. ✓ Increased the anti-inflammatory and fiber-fermentative *Blautia* genus, which belongs to this *Lachnospiraceae* family. ✓ Reduced important pro-inflammatory bacterial populations, such as those of the genus *Desulfovibrio* and *Bilophila*. ✓ Significantly reduced the number of hyperplastic Peyer’s patches in the small intestine mucosa. ✓ Increased cecum weight (the whole cecum, including cecum containing feces) as the cecum works as a bioreactor, where microbiota flourish in the presence of fermentable fibers, such as inulin, increasing the weight of this organ and its contents. ✓ Reduced (49.9%) the number of colon polyps.
[27]	Research Rat where CRC was generated using AOM/DSS	GOSLu (galacto-oligosaccharides derived from lactulose) 2 g per rat		✓ Significantly reduced populations of pro-inflammatory bacteria families and species, and significant increases in interesting beneficial populations, such as *Bifidobacterium*. ✓ Allowed diverse SCFAs. In this study, a 56.9% increase in the caecum production of propionate was clearly observed, in a statistically significant way. ✓ There was a statistically significant reduction in tumor number and area. ✓ Increased *Bacteroidetes* and reduced *Firmicutes* populations and a reduction in *Desulfovibrio* genus (*Desulfovibrionaceae* family) populations. ✓ Increased *Phascolarctobacterium* genus (which belongs to the *Acidaminococcaceae* family, a *Firmicute*).
[29]	Research Rat with induced carcinogenesis	Jabuticaba [*Myrciaria jaboticaba* (Vell.) O.Berg] seeds, a native berry from a multi-stemmed tree indigenous to Brazil isthe richest source of phenolic compounds, such as anthocyanins and ellagic acid	Yogurt *Streptococcus salivarius* subsp. *thermophilus* and *Lactobacillus* *delbrueckii* subsp. *Bulgaricus*	✓ Increased *Bacteroidetes* abundance and a decreased *Firmicutes* abundance. ✓ Reduced bacterial metabolizing enzymes-β-glucoronidase, β-glucosidase, β-galactosidase, mucinase, and nitroreductase, thus reducing colonic tumor incidence; inhibited the growth of *Bacteroides*, *Clostridium*, and *Propionibacterium* spp., thus enhancing the abundance of *Lactobacillus* and *Bifidobacterium* species. ✓ Ellagic acid and ellagitannins are the main compounds in LJE, can be degraded in the gut producing urolithins, which in turn has shown to inhibit the proliferation of cancer cells by regulating cellular activities and signaling pathways and to decrease the activation of proinflammatory cytokines in the gut. ✓ Equalized the biodiversity of bacteria by changing the abundance of *Firmicutes*, *Bacteroidetes*, and *Proteobacteria* phyla in rats.
[30]	Research carcinogen-induced rat model	Djulis (*Chenopodium formosanum*) is a native cereal crop 10% djulis in the experimental diet is equal to 44 g djulis for a 60 kg human per day		✓ Inhibited the progress of CRC by regulating the colonic secretion of mucins. ✓ Had an inhibitory effect on the progression from primary to advanced precancerous lesions during colon carcinogenesis. ✓ It is speculated that Djulis could provide prebiotic dietary fiber for promoting the growth of *L. acidophilus* in the intestine. COX-2 expression was reduced. It may suppress colon carcinogenesis via an inflammation-associated pathway. ✓ Reduced distal Aberrant Crypt Foci (ACF). ✓ The low number of Mucin-Depleted Foci (MDF). ✓ Decreased expression of Bcl-2 in all Djulis-treated groups.

**Table 3:** Prevention of Colorectal cancer (CRC) using various prebiotics in cell lines.

Reference	Type of Study	Prebiotic	Probiotic	Mechanism of Action
[31]	Research Cell growth-inhibitory activity in DLD-1 cells and WirDr cells	fructo-oligosaccharides (FOS)	*Bifidobacterium longum* (BB536-y)	✓ Intake of BB536-y with FOS was associated with a higher *Bifidobacterium* detection rate than that of BB536-y alone. ✓ The contents of butyric acid, isobutyric acid, and acetic acid, namely of SCFA, were also decreased. ✓ Analysis of the results of culture of DLD-1 cells and WirDr cells in the presence of butyric acid, isobutyric acid, and acetic acid revealed that each of the substances showed significant cell growth-inhibitory activity, with the activity being the highest for butyric acid, followed by that for isobutyric acid and acetic acid.
[32]	Research HT29 Cell line	Soluble dietary fibre extracted from plantain inflorescence (PIF) ^1^		✓ Promoted the growth of *L. casei* and *B. bifidum* which indirectly inhibits the growth of pathogenic strains and protects the intestine. ✓ Rich in SCFA particularly that obtained from fermentation by *B. bifidum*. ✓ The study particularly identified up-regulation of one of the key apoptotic inducing protein—Apoptosis-inducing factor, mitochondria-associated. Thus, it is evident from the experimental results that the fermentation supernatant contains SCFA which induces ROS mediated apoptosis in HT29 cells.
[33]	Research Cell lines and MTT assay ^2^	Polysaccharide fraction from mushrooms *Cantharellus cibarius*		The prebiotic potential was revealed in relation to *Lactobacillus* strains. Crude polysaccharides were found to inhibit the proliferation of colon cancer cells with the simultaneous absence of toxicity towards normal cells.

^1^ Rich source of dietary fiber and polyphenols exhibit anticancer potential in HT29 colon cancer cell. ^2^ Antiproliferative activity (MTT assay). Cell proliferation was assessed by means of the MTT assay, in which the yellow tetrazolium salt (MTT) is metabolized by viable cells to purple formazan crystals.

**Table 4:** Prevention of Colorectal cancer (CRC) Using Various Prebiotics in Human Clinical Trials.

Reference	Type of Study	Prebiotic	Probiotic	Mechanism of Action
[35]	Research Human, A randomized, double-blind, no-treatment parallel control, clinical trial study involving 140 perioperative patients (90 men and 50 women, aged 40–75 y)	30 g prebiotic supplement (Hangzhou Niuqu Biotech Co., Hainengbo, China) containing fructooligosaccharide (25%), xylooligosaccharide (25%), polydextrose (25%), and resistant dextrin (25%)		✓ Improved serum immunologic indicators (significantly increased IgG and IgM levels preoperatively. Postoperatively the supplementation enhanced the levels of IgG, IgA, total B lymphocytes (CD19+), and suppressor/cytotoxic T cells (CD3+CD8+). ✓ Prebiotics increased the level of transferrin as prebiotics relieve the inflammatory reaction of the body, resulting in increased transferrin level. ✓ Altered the intestinal microbial community at the at the genus level: a decline in *Bacteroidetes* in the prebiotic/pre group. The abundance of *Bifidobacterium* and *Enterococcus* increased significantly in the prebiotic/pre group. It is proposed that *Bifidobacteria* prevent against CRC by regulating intestinal microbiota, enhancing host immune response, and degrading potential carcinogens. ✓ Increased the abundance of intestinal opportunistic pathogens and harmless strains of *Escherichia* species.
[36]	Cohort postmenopausal women in the United States	Prebiotic fiber supplements categorized as soluble or insoluble		The findings do not support use of prebiotic supplements to reduce risk of colorectal cancer or colorectal cancer–specific mortality among postmenopausal women.
